# Recent change in spatial distribution of the European flat oyster (*Ostrea edulis*) inferred from field data and empirical models of living oysters and empty shells

**DOI:** 10.1002/ece3.8925

**Published:** 2022-05-15

**Authors:** Per Bergström, Linnea Thorngren, Mats Lindegarth

**Affiliations:** ^1^ 3570 Department of Marine Sciences – Tjärnö University of Gothenburg Tjärnö Sweden

**Keywords:** conservation, distribution, management, modeling, *Ostrea edulis*, Shell

## Abstract

Marine coastal areas are increasingly affected by human activities resulting in changes in species and habitat distributions. Understanding these patterns and its causes and consequences is important for conservation and restoration of such changing habitats. One habitat that has been heavily affected by human use are the North Sea oyster beds which once were abundant but have lost large parts of its coastal distribution due to overexploitation. Based on data of living and dead assemblages of *Ostrea edulis* collected using video transects, we used an ensemble modeling technique to model and predict current and recent distribution of *O*. *edulis* along the Swedish west coast where its distribution is, in relative terms, still rather unaffected. We could detect a recent change in the distribution of *O*. *edulis* along the coast which to a large extent could be attributed to a change in depth distribution, suggesting that the population of *O*. *edulis* have a slightly shallower distribution today than in the past. Although a potential mismatch between living and dead assemblages, caused by a complex combination of biological and environmental conditions, needs to be considered in the interpretations drawn, it may be a way around the lack of suitable background data in management decisions. This provides important information for management and conservation of the native oyster beds. Furthermore, this study illustrates a method for identifying recent changes in species distribution using dead assemblages of bivalves.

## Graphical abstract

Based on data of living and dead assemblages of *Ostrea edulis* collected using video transects, we used an ensemble modeling technique to model and predict current and recent distribution of *O. edulis* along the Swedish west coast. We could detect a recent shift in distribution of *O. edulis* along the coast which to a large extent could be attributed to a change in‐depth distribution suggesting that the population of *O. edulis* have a slightly shallower distribution today than in the past.

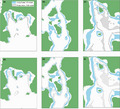

## 1 INTRODUCTION

As human population have grown, so have the pressure on coastal environments. Humans have long been dependent on the coastal zones and the services its ecosystems provide (Mehvar et al., 2018; Neumann et al., 2015). Thus, human activity has highly modified marine ecosystems in temperate regions and, in combination with effects of global climate changes, many of these ecosystems are far from their natural and original status (Halpern et al., 2008, 2015), and the speed at which the pressures on the ocean increases are growing (Halpern et al., 2019). One of these intensively used areas is the North Sea which, in a relatively short time, has lost almost all its offshore and large part of its coastal oyster grounds due to overexploitation (Beck et al., 2011). Once abundant and ecologically important, reef habitats have vanished from the ecosystem and today, beds of the European flat oyster (*Ostrea edulis)* are rare or absent from most of their natural range (Airoldi & Beck, 2007; Haelters & Kerckhof, 2009; Laing et al., 2005). These reef structures are now protected and should, according to the Habitats Directive, be preserved and/or restored to favorable conservation status. Oyster reefs are also listed as threatened by the OSPAR convention (Haelters & Kerckhof, 2009) and its signatory states should protect, maintain, and expand its remnant oyster populations (Kerckhof et al., 2018) while also trying to restore previous oyster area (Farinas‐Franco et al., 2018; Gercken & Schmidt, 2014; Smaal et al., 2015). Oyster reefs produce a variety of important and valuable ecosystem services: improved water quality, increased nutrient uptake, complex three‐dimensional structure which provide habitats, food, and protection for a large number of species, as well as providing a valuable food and economic resource for humans (Gerritsen et al., 1994; Grabowski et al., 2005; Nelson et al., 2004).

Restoration efforts of *Ostrea edulis* are currently restricted to the few remnant populations which is already heavily impacted by historical exploitation. Since the “natural” state of oyster beds have often been defined at the time when the protective area was designated, the baseline upon which changes are measured and on which management is determined is often already an affected state which might not reflect the past conditions. Assessing fully natural conditions is often difficult as impacts most often pre‐date detailed documentation of the areas. Thus, conservation, restoration, and management are often based on incomplete knowledge and reference conditions.

However, due to the properties of oyster beds inherited by the fact that the oysters produce relatively thick and stable shells which remain long after the animal itself dies, dead shell assemblages is considered one of the most crucial components of a healthy oyster reef and is thus considered a main component in oyster reef restoration efforts. Comparison of living and dead assemblages offers possibilities to study how processes of settlement and decomposition influence the preservation of species in fossil record (Kidwell, 2013), although caution is needed when interpreting results as anthropogenic impacts are known to cause mismatch between living and dead assemblages (Kidwell, 2013; Kidwell & Tomasovych, 2013). However, the dead shells also offer another less studied opportunity, the potential to back track recent changes in the spatial distribution of oyster beds. Applications of dead assemblages of organisms in investigations of ecological changes have been scarce, but these studies have demonstrated that the distribution of dead organisms can function as a long‐term record of changes in community structure and function (Liversage et al., 2020). For example, Kidwell (2007, 2008) investigated community changes in response to anthropogenic activities (e.g., fishing) using dead assemblages.

Using dead assemblages for tracking short‐ and long‐term changes in spatial distribution of the living community over time can potentially offer an important opportunity to follow changes in ranges due to, among others, human pressure when time series of a species large‐scale spatial distribution is lacking. How far back you can go depends on species and local biological and environmental processes. For oysters, this is limited by the destruction and dissolution of its shell which in turn depends on a combination of biological, geochemical, and sedimentary factors; the same processes that ultimately control the rate of loss from natural reefs. Estimates of half‐life times for oyster shells vary from a few years to a few decades. Powell et al. (2006) calculated the half‐life time for Eastern oysters (*Crassostrea virginica*) to between 1 and 20 years while Waldbusser, Steenson, et al. (2011) estimated it to be up to 40 years. The shells might also be transported away from its origin contributing to the uncertainty in the time span available. However, this transport is not well known and thus difficult to account for, and with rather heavy shells, of oysters, is restricted to more exposed areas. With shell‐boring organisms generally considered to be the primary reason for oyster shell degradation (Carver et al., 2010), longer half‐life times are expected in areas scares of such organisms.

With humans increasingly altering the distribution of species by modifying habitat, changing global climate, and introducing new species (Mack & Lonsdale, 2001; Parmesan, 2006; Tilman & Lehman, 2001), successful management of biological resources, such as oyster or mussel beds, depends on our ability to predict the potential spatial distribution of range‐changing species and to understand the forces that limit their distribution. Combining knowledge of distribution of living and dead oysters (or other bivalves) with species distribution modeling (SDM) and geographic information system (GIS) offers the possibility to predict and map current spatial distribution (based on living oysters) and former recent areas (based on dead shells), which can be used in conservation to identify areas of special interest for restoration and conservation project.

The aim of the study was to investigate similarities and differences in predictive models and predicted distribution of adult living *Ostrea edulis* densities >1 ind.m^−2^ and sites with high abundances (>1 ind.m^−2^) in dead shells to identify and evaluate recent changes in the spatial distribution of the population along the Swedish west coast. We also demonstrate a potential new method for predicting short‐term changes in species distribution based on species distribution modeling technique and field data on living and dead shells. For these purposes, we generated two models, using a similar approach as Bergström et al. (2021), to (a) predicted distribution of high‐density areas of living oysters (LO), and (b) predicted distribution of areas with high densities of empty oyster shells (EOS) in the absence of high densities of living oysters. These were used to identify differences between areas with living high densities of *O*. *edulis* and areas in which the abundances of shells indicate a recent, but no longer, high abundance of living oysters, and finding places with either living or dead high densities. We also used the results to discuss potential explanations to changes in distribution and potential distinguishing characteristics of sites which previously have had high densities (i.e., those with a lot of dead oysters) but which now have none.

## 2 MATERIALS AND METHODS

### 2.1 Study area and species distribution data

**FIGURE 1:**
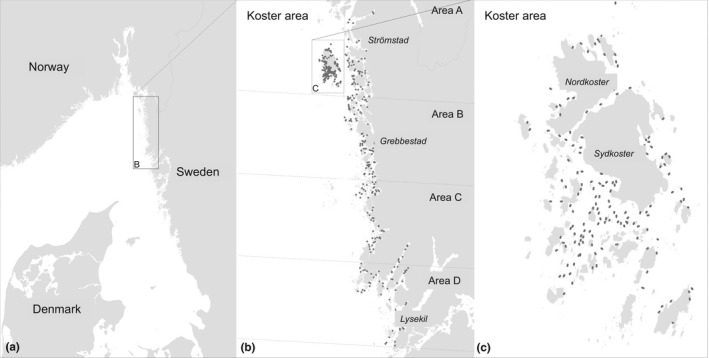
Overview of study area (a) and sample intensity (b) with close‐up on the most intensive sample areas (c) within the Kosterhavet National Park

This study was carried out in the northern part of the Swedish west coast bordering Skagerrak (Figure 1). This area is dominated by a mixture of rocky and sandy shores spread across an extensive archipelago with large number of islands, of which the inner parts may be ice covered during the winter, and it host the vast majority of the Swedish native flat oyster population. A dataset of abundance of living *Ostrea edulis* and dead shells was extracted from the studies performed by Thorngren et al. (2019, 2017). Thorngren et al. (2019) visited a total of 452 randomly selected locations in the study area during a 2‐year period (2013–2014). Sampling was restricted to a maximum depth of 10 m and stratified into three depth strata (0–3, 3–6, and 6–10 m) and to areas classified as moderately exposed or less according to the classification of wave and wind exposure (Naturvårdsverket, 2006). In each of these locations, two 20‐m‐long, 0.8‐m‐wide video transects were filmed, at a speed of approximately 0.4 knots, using a downward‐facing high‐definition camera mounted 50 cm from the sea floor to a towed sledge. The video films were then analyzed according to Thorngren et al. (2017) with number of adult sized “living,” “possibly living,” and “dead” oyster recorded. From the results presented in Thorngren et al. (2017), which investigated small‐scale variability, we know that the distribution of living *Ostrea edulis* is patchy at the level of individual transects. But, having long enough transects, the effect of this patchiness is negligible in this study. This sampling method was validated in Thorngren et al. (2017) by comparing video data, from filmed transects, with field measurement made by snorkeling in the same transects. The overall correct classification rate using this method was found to be 0.81, while the significant variability between observers were less than 1%. Detailed results are presented in Table 2 and the associated text in Thorngren et al. (2017).

Although often a density of 5 ind. m^−2^ is conventionally used in conservation purposes, e.g., OSPAR, to define oyster beds (Haelters & Kerckhof, 2009), we used a more inclusive level of 1 ind. m^−2^ as definition of high‐density areas in this study. The rationale behind this is the observation made by Thorngren et al. (2019) and Bergström et al. (2021), which showed that high‐density areas using the OSPAR definition would correspond to roughly 1% of the visited sites while the one selected here would include roughly 5% of the sites and 85% of the oyster population. Thus, the selected level of 1 ind. m^−2^ provides a better separation between those sites that contribute most to the total population from the rest while at the same time provide a solid base for modeling. For further details on experimental design and sampling, see Thorngren et al. (2019, 2017).

### 2.2 Environmental data and pre‐modeling analysis

Three environmental predictor variables (depth, salinity, and exposure) were selected for the modeling. The candidate predictors were based on the general relevance for species distribution in marine environment, their availability as full covering raster layers for the investigated area, and the results obtained by Bergström et al. (2021). Wave exposure data from the Naturvårdsverket (2006) were corrected for depth according to Bekkby et al. (2008) to generate depth‐attenuated exposure information, which should better reflect the conditions at the bottom. The same unpublished minimum salinity layer, interpolated from more than 20,000 measurements retrieved from the ICES database [https://www.ices.dk/data/dataset‐collections/Pages/default.aspx], described by Bergström et al. (2021) were used for this study while depth was measured in field. Pre‐modeling, the selected explanatory variables were tested from correlation using variance inflation factor analysis. This is a simple approach to identify collinearity among predictor variables and those who obtain a high value in the calculations are removed. Although arbitrary, a value of 5–10 is normally considered high collinearity (Akinwande et al., 2015; Hair et al., 1995; Kline, 1998), here we used a more conservative level of 3. These initial analyses showed that the tree environmental variables were largely uncorrelated (VIF value below 3) within the sampled data and were thus considered sufficiently uncorrelated for use in modeling.

### 2.3 Modeling algorithms

We fitted an ensemble species distribution model based on nine commonly used algorithms: four machine learning methods, Artificial Neural Networks (ANN, Ripley, 1996), Classification Tree Analysis (CTA, Breiman et al., 1984), Generalized Boosted Models (GBM, Ridgeway, 1999), and Random Forest (RF, Breiman, 2001); four regression‐based methods, Generalize Additive Models (GAM, Hastie & Tibshirani, 1990), Generalized Linear Models (GLM, McCullagh & Nelder, 1989), Multivariate Regression Splines (MARS, Friedman, 1991), and Flexible Discriminant Analysis (FDA, Hastie et al., 1994); and one envelope‐style method, Surface Range Envelope (SRE, Busby, 1991). In this study, the BIOMOD2 package (Thuiller et al., 2016) for R software (R Core Team, 2019) and its related packages were used for all the selected algorithms. The default settings for modeling options in BIOMOD2 were used which do not include interactions for the regression‐based methods. The reason for this was that incorporating interaction terms would quickly increase the number of effective variables. A consensus ensemble approach was applied using the BIOMOD2 platform models generated by the selected individual models. This approach should in theory offer a more robust, less noisy, prediction for the potential and realized distribution of *Ostrea edulis* than single algorithm models. Models were built using a 100‐fold cross‐validation and randomly splitting the data into training (70%) and test data (30%) for respective model calibration and testing. This splitting procedure permit evaluation of model accuracy and predictive performance when data are non‐independent.

### 2.4 Modeling evaluation

The performance of the models (i.e., strength of agreement among distribution data and each model) was assessed using the area under receiver curve (AUC, Hanley & McNeil, 1982) and true skills statistics (TSS, Allouche et al., 2006; Liu et al., 2005) together with sensitivity (percentage of good presence predictions) and specificity (percentage of good absence predictions). AUC is threshold independent and evaluates both false‐positive error rate and the true‐positive rate in order to obtain a measurement of the model accuracy while TSS takes both omission and commission errors into account (Allouche et al., 2006) maximizing the sum of sensitivity and specificity. AUC range from 0 to 1 with values below 0.5 representing models that is not better than random and a value of 1, a highly accurate model (Scarnati et al., 2009). Although AUC has been highly criticized in some studies (Austin, 2007; Jimenez‐Valverde, 2012; Raes et al., 2009), it is still the most commonly use measure to assess model accuracy and therefore considered a useful measure for this study. TSS range from −1 to 1 with values above 0.6 considered useful (Coetzee et al., 2009). To ensure an accurate model prediction, only individual models above a critical TSS (0.6) value were implemented in the final ensemble model, further the included models were weighted based on their TSS values. This was done because weighted means have been suggested to have the best performance of the ensemble methods available (Marmion et al., 2009).

Functional relationship between the explanatory variables and the response, either occurrence of high‐density (>1 ind. m^−2^) living oysters (LO) or high‐density (>1 ind. m^−2^) dead shells (EOS), was further explored using partial dependence plots. The predictors that most strongly influence the model were determined using variable importance assuming that the most important variables are the ones with a relative importance above the mean of the predictor variables within each subset. Variable importance was estimated using the built‐in function in the BIOMOD2 package (Thuiller et al., 2016), which is based on shuffle a single variable of the given data, making model predictions using this “shuffled” data and then computing the Pearson correlation between reference and “shuffled” predictions returning a value between 0 and 1 were the higher value the higher importance of that variable. One limitation of this method is that it does not account for interactions between different variables.

### 2.5 Prediction and post‐modeling analysis

To evaluate the potential recent loss of high‐density oyster habitats, after the final ensemble models were created and evaluated for variable importance and predictive power of the environmental variables, they were applied to high‐resolution (15 × 15 m) raster layers of the environmental variables to generate probability maps of occurrences (living oysters and dead shells, respectively). These probability maps were translated into presence–absence distribution using the threshold (cut‐off, optimized based on a data‐driven approach using Youdens index) calculated by BIOMOD2 where all areas (15 × 15 m raster cells) with a predicted probability above the threshold (>cut‐off) grouped into “present,” whereas lower suitability values were grouped into “absent.” The observed results were then analyzed visually to identify areas of high interest for further studies on changes in the distribution of living oysters along the Swedish coast. Since areas based on cut‐offs optimized using Youdens index (minimizing the total error, that is, false negatives plus false positives) for each model are not easily comparable between models, we also used a more “decision‐analytic approach” where the same cut‐off values were used for both models. This allowed us to estimate areal extent of LO and EOS plus overlapping areas, under conditions where the criteria for classification are equal for LO and EOS models.

## 3 RESULTS

### 3.1 Pre‐modeling results

Of the total 452 sites investigated, 59 sites had high densities (>1 ind. m^−2^) of living oysters, dead shells, or both. Of these 59 sites, 52 had high densities of dead shells and 27 had high densities of living oysters. Thirty‐two sites had only high densities of dead shells while only seven sites displayed high densities of living oyster without at the same time having high densities of dead shells. The remaining 20 (of the total 27) sites with high densities of living oysters also had high densities of dead shells in its assemblages. The average depth of the sampling sites was 4.41 m (±2.65) while the mean salinity for the sites was 17.3 (±4.0). After recalculating the exposure accounting for depth at the individual sites, the observed average depth‐attenuated exposure was 13302 (±38,231).

**FIGURE 2:**
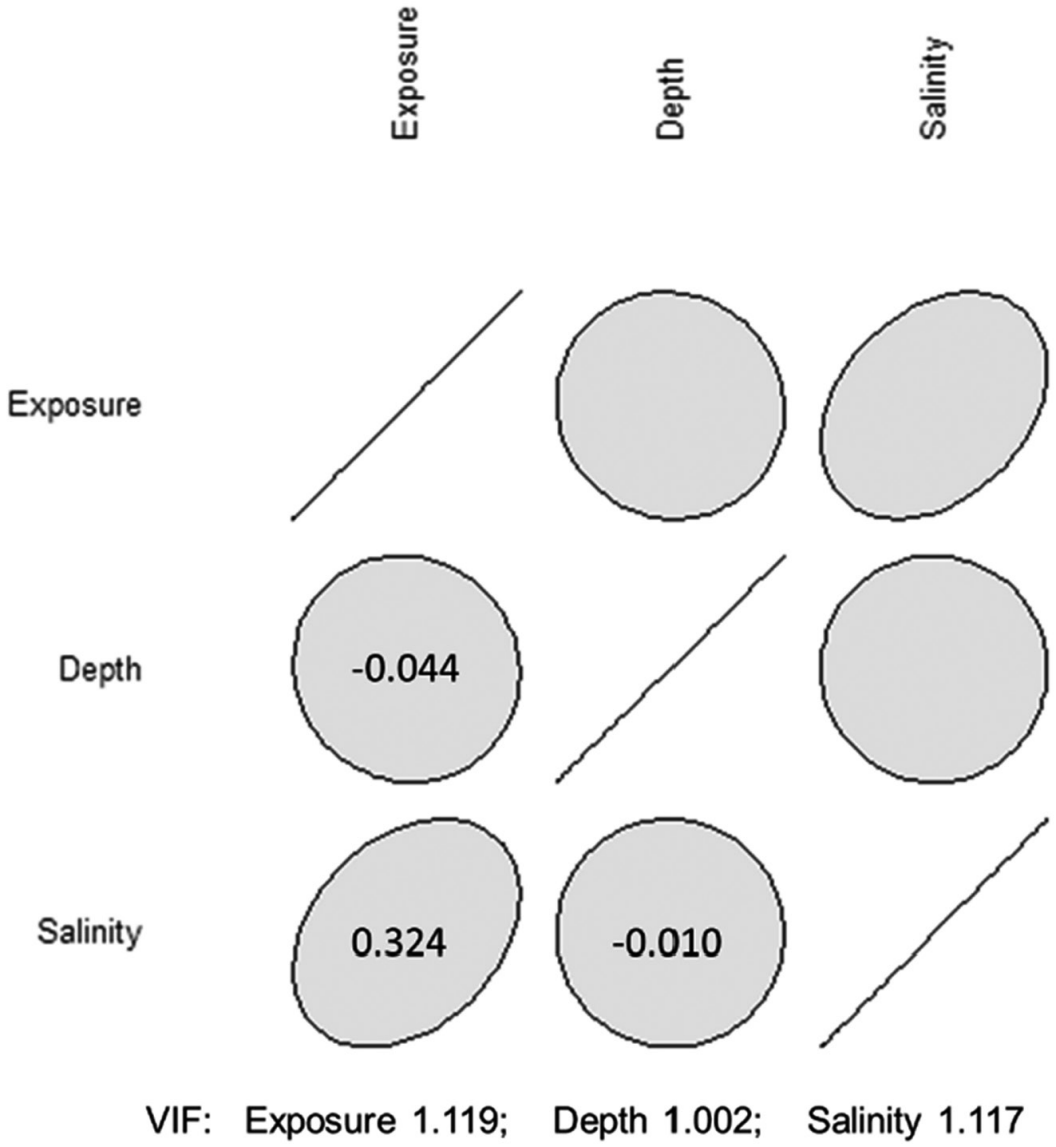
Correlation and variation inflation factor among the three environmental predictors in the model

Both analysis on variance inflation factor (VIF) and the correlation plot showed no strong relationship among the predictors, with all VIF values just above 1 (i.e., no multicollinearity among predictors) and only weak correlations between variables (Figure 2). The strongest correlation was found between salinity and exposure (0.32).

### 3.2 Model performance and variable importance

**FIGURE 3:**
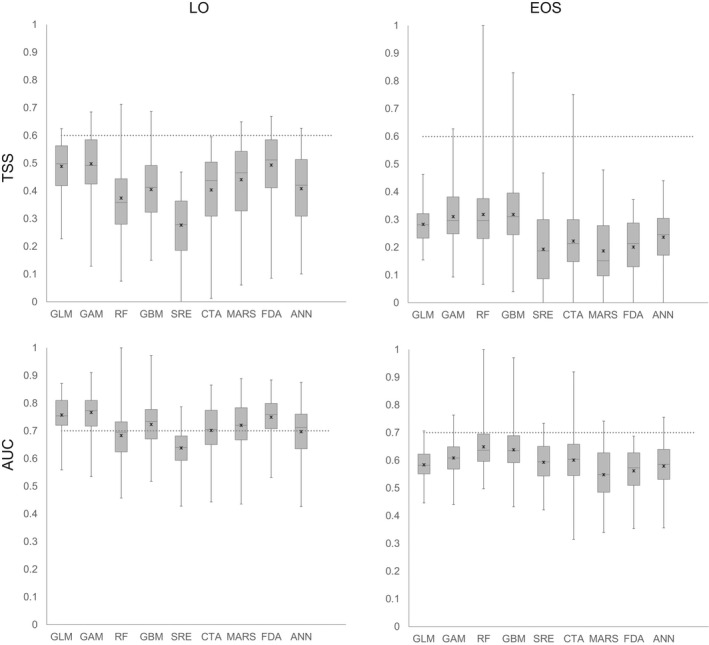
Model performance (TSS and AUC) of the nine methods for predicting distribution of high densities of (a) living oysters (LO) and (b) empty oyster shells (EOS). The models from left to right in the diagrams; GLM (Generalized Linear Models), GAM (Generalized Additive Models), RF (Random Forest), GBM (Generalized Boosting Model), SRE (Surface Range Envelope), CTA (Classification Tree Analysis), MARS (Multiple Adaptive Regression Splines), FDA (Flexible Discriminant Analysis), and ANN (Artificial Neutral Networks). Dotted line = standard limit for good models, AUC = 0.7, TSS = 0.6. Performance of the final ensemble models is AUC = 0.94 and TSS = 0.76 for living oysters (LO) and 0.999 and 0.990, respectively, for dead oysters (EOS)

The predicted distribution maps of both living *Ostrea edulis* and empty *O*. *edulis* shells resulted from ensemble forecasting parameterizing TSS >0.6 for 900 individual models (100 runs of nine methods). Figure 3 shows boxplots for TSS and AUC scores of the 900 individual models. The best performing techniques on average were GLM, GAM, FDA, and Mars, which all performed at a similar level (Figure 3). Random Forest (Rf) models displayed the largest variation, i.e., largest span in TSS and AUC, respectively, for individual models within each method but were on average (mean TSS/AUC of all individual models) among the worst performing models (together with SRE models) when including living oysters, even though some of the absolute best performing individual models were also found among the RF models. For the presence of high densities of empty shells (EOS), the RF technique performed better (i.e., higher TSS and AUC values) than the other methods (Figure 3). The individual models generally performed better for living oysters than for models including empty shells but the ensemble models performed better when focusing on empty shells (Figure 3, Table 1).

**TABLE 1:** Model performance (AUC and TSS) of ensemble models on oyster densities above 1 oyster m^−2^ for living assemblages and assemblages of only dead oysters

Model	AUC	TSS	Sensitivity		Specificity		Cut‐off	
			AUC	TSS	AUC	TSS	AUC	TSS
Living oyster (LO)	0.94	0.76	88.89	88.89	87.77	87.06	101.5	100
Empty shells (EOS)	0.999	0.990	100	100	99.29	99.05	323.5	322

Upon building the ensemble models, GL, RF, GAM, GBM, MARS, FDA, ANN, and CTA models were included in the ensemble model of living oysters while RF, GAM, GAM, and CTA models were used for the model for dead assemblages. The two ensemble models, one for living oysters (LO) and one for dead assemblages (EOS), were generally very accurate with AUC scores >0.9 for both LO and EOS models and TSS scores between 0.76 and 0.99 (Table 1). The models were good at identifying true presences within the dataset, showing sensitivity values above 88%, while at the same time being able to identify true absences in more than 87% of the cases. When including empty shells in the modeling, specificity and sensitivity increased compared to the LO model (Table 1).

**FIGURE 4:**
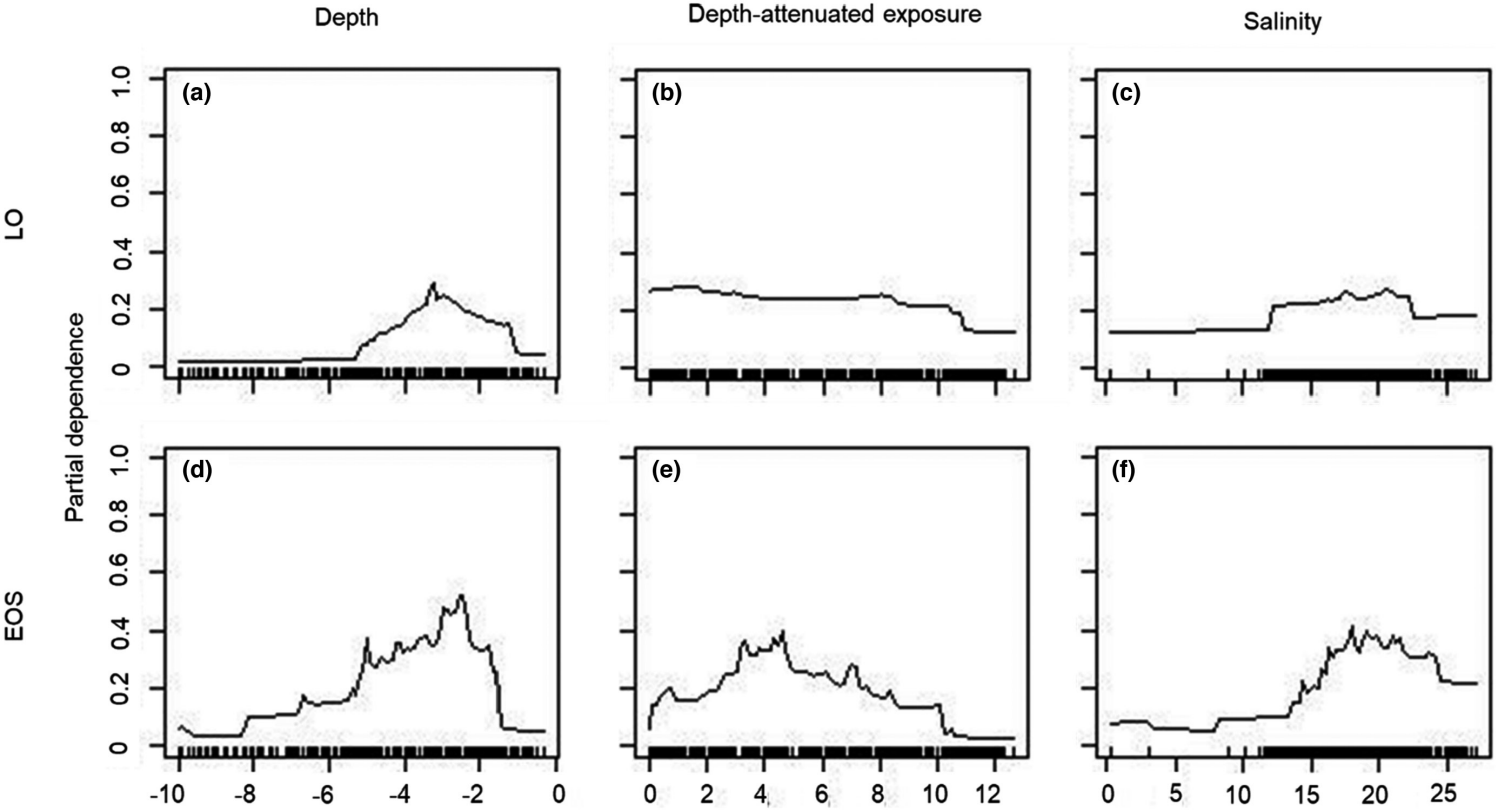
Partial dependence plots for living oysters (LO; figures a–c) and dead shells (EOS; figures d–f) showing the partial dependence of depth (a, d), log(depth‐attenuated exposure) (b, e), and salinity (c, f)

Analysis of variable importance for the ensemble models shows that depth has the strongest influence on the ensemble model for both models (LO and EOS). For the LO model, depth was followed by depth‐attenuated exposure and salinity being the least importance variable, while for the EOS model, exposure and salinity were in the reverse order of importance (Table 2). However, in the model including empty shells (EOS), the difference between the importance of depth and the other predictors is smaller and depth and salinity had almost the same importance. Although more important for EOS model, the partial plots (Figure 4), showing the functional relationship between environmental predictors and the probability of occurrence showed the same general pattern for both EOS and LO models with regard to salinity and exposure (Figure 4b,c,e,f). However, with a slightly more distinct peak in partial dependence of exposure for EOS models than LO at the middle of the investigated exposure range.

**TABLE 2:** Variable importance for the two different model types: living (LO) and dead empty shells (EOS) oyster assemblages

Model	Depth	Exposure	Salinity
LO	0.837	0.241	0.136
EOS	0.588	0.386	0.551

### 3.3 Prediction and post‐modeling results

**FIGURE 5:**
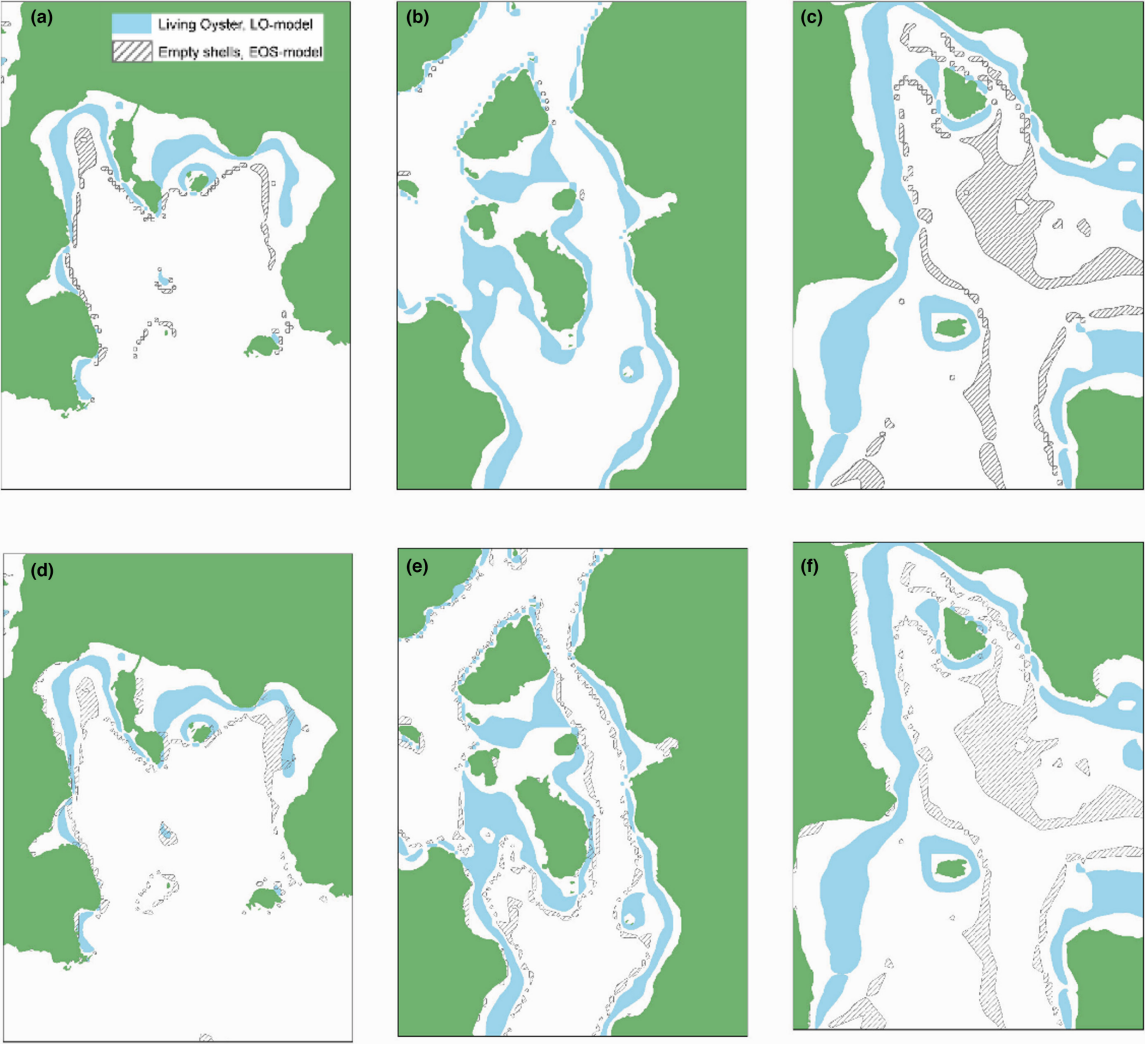
Examples of predicted distribution of high densities of living oyster and empty shells showing a general pattern of changed distribution of living oysters. The pairs of maps (a–d, b–e, and c–f) represent the predictions using optimal cut‐offs (a, b, and c using Youdens index) and a more “decision‐analytic approach” using equal cut‐offs (d, e, and f). Land is presented in green, Predicted areas of high densities of living oysters in blue and high densities of dead assemblages in striped

**FIGURE 6:**
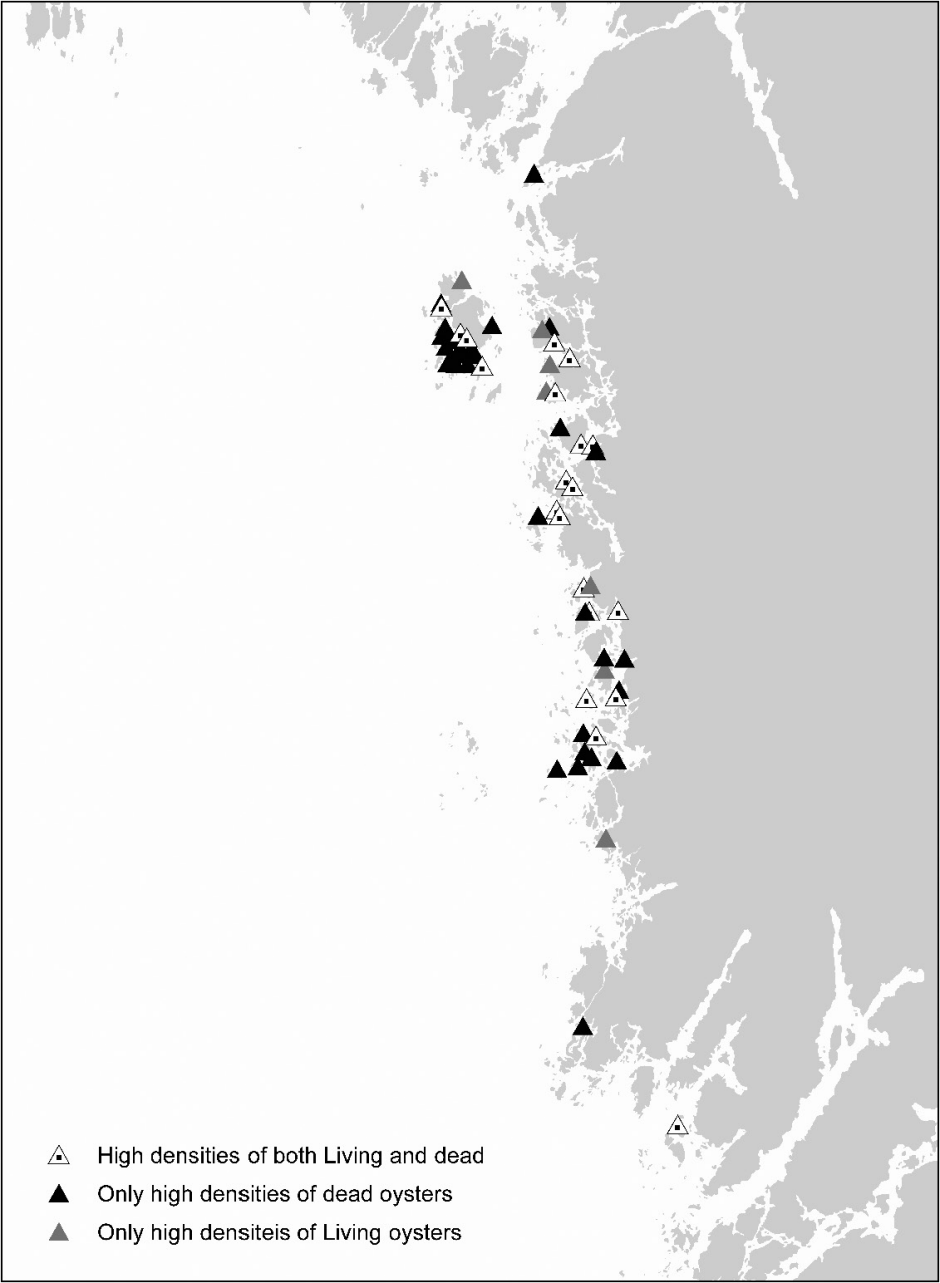
Sites with observed high densities of living *Ostrea edulis* (grey triangles), dead (black triangles), and sites containing both high densities of both living and dead assemblages (white triangles with black dot)

As illustrated by the partial plots in Figure 4 and the examples in Figure 5, there seem to have been a change in the spatial distribution of oysters toward slightly shallower areas in recent times. This pattern was independent of whether optimized cut‐off (Youdens index; Figure 5a–c) or whether a criteria of equal threshold values were used (Figure 5d–f). Further visual analysis of the observed pattern and the distribution of sites with observed occurrences of high densities of living and dead assemblages, respectively, showed that it is mainly in the northern part of the study area where the high‐density sites area was found with only a few sites in the southern part of the area displaying high densities of either living or dead assemblages (Figure 6). Furthermore, although sites of only dead assemblages were found throughout the entire northern part of the study area, the highest frequency of sites with only dead assemblages was observed in two areas close to the northern and southern limits of the “high‐density area,” respectively. The placement of these areas with dead assemblages indicates that the total spatial distribution of high‐density areas has shrunk slightly (Figure 6). Figure 6 also illustrates that there seem to be slightly less occurrences of sites with only dead assemblages in more sheltered areas compared to more exposed areas.

In order to estimate the overall areal extent and map the locations of living oysters (LO) and empty shells (EOS), we utilized predictions of high densities of living oyster and empty shells. The areal extents were predicted using both model‐specific optimized cut‐offs (Youdens index) and the decision analytic approach of equal cut‐offs. These analyses showed that the predicted areal extent of living oysters and empty shells was largely sensitive to cut‐off values (Table 3, Figure 5). Using model‐specific optimization to minimize total error, we obtained an overlap of only 3% between living oysters and empty shells of the total predicted area while living oysters covered 75% of the area. Instead, if using equal sized cut‐offs, the overlap was 10% and the dead assemblages made up 45% of the total predicted area (Table 3). The corresponding observed values were 34% overlap, 12% areas with only living assemblage, and 54% of the total observed sites with high densities having only high densities of empty shells.

**TABLE 3:** Comparison of occurrence of high‐density areas with living oysters (LO), empty oyster shells (EOS), and both LO and EOS for observed and modeled data

Type	Observed		Modeled (Youden)		Modeled (equal probabilities)	
	# Of sites	%	Predicted area (ha)	%	Predicted area (ha)	%
LO	7	12	1408	75	1214	46
LO & EOS	20	34	62	3	256	10
EOS	32	54	408	22	1194	45
Total	59		1877		2664	

Note

Cut‐off values for models optimized using Youdens index are LO = 0.1 and EOS = 0.3, and for equal probabilities, LO = EOS = 0.1.

## 4 DISCUSSION

For meaningful targets for conservation and restoration, it is vital with baseline information on the status of the community/species before a recent and/or rapid change (NRC, 2005). However, well‐defined historical data are scarce and, if present, often local. Thus, alternative sources of information are needed if recent changes are to be investigated. Although recent analyses have shown that the Swedish population of the European flat oyster is still relatively healthy with densities and population size being rather substantial compared to populations at other European locations (Thorngren et al., 2019; for other examples of remaining strong populations of *Ostrea edulis*, see Allison et al., 2020, Lown et al., 2020), it is still affected. Using an ensemble modeling approach and field data of presence and abundance of living *Ostrea edulis* and empty shells, this study demonstrates an example of how empty shells of living organisms can be used for prediction of recent (8–15 years) changes in spatial distribution. The results found in this study also provide important information on the current distribution of *Ostrea edulis* and how the distribution has changed during recent years toward slightly more shallow areas. We also note that exposure and salinity are more important in determining the distribution of empty shells than they are for explaining the distribution of living oysters. It gives insights into the development of tools for management of oyster beds and for planning and management of the coastal environment.

For environmental assessment, the mismatch between living and dead assemblages is not perfect, as there are some overlap between dead assemblages and pristine areas and the assemblages of dead shells may represent a range of time spans depending on local biological and environmental conditions. Often these assemblages of dead shells include a majority of relatively recent deaths and a long tail of rare shells from individuals that died at increasingly earlier time. Given these time spans, the distribution of dead shells may not be a perfect indicator of present or past habitats, restricting the interpretations and conclusions that can be drawn from the mismatch in distribution between living and dead assemblages (Powell et al., 2017). Additionally, potential accumulation of transported shells further complicates the interpretation (Callender et al., 1992; Miller, 1988; Zenetos, 1990) as will the tendency of species not to be distributed in all suitable habitats during all time (Levinton, 1970; Powell et al., 1986). Thus, the spatial distribution of dead shells and living animals may not always be a suitable indicator of present and/or past habitat. However, given the lack of background data available in most areas and the urgent need for baseline data to support management and conservation decisions, the use of living versus dead assemblage's might potentially be the only way to provide evidence of recent changes in distributional patterns in the absence of widespread baseline information. Tomašových and Kidwell (2009) showed that death assemblages have the same ability to capture environmental gradients as living assemblages, further supporting the idea of using dead assemblages to track recent changes in species distributions. This potential is to a large degree unaddressed in marine environments. Furthermore, in cases when historical and contemporary knowledge on the vertical distribution of regional species populations is inadequate, as for the Swedish *Ostrea edulis* population, the use of methods utilizing dead assemblages might be the only way to obtain some scientifically based baseline information for future management of the population. As in the case of *Ostrea edulis*, heavier shells require a much higher water movement energy for transportation, making them more suitable for utilization of recent changes in spatial distribution than lighter shells such as blue mussel (*Mytilus edulis*) shells which are more easily transported away from its origin.

The observed changes in depth distribution based on empty shells versus living oysters could, in theory, potentially be an effect of higher fishing pressures in deeper waters. However, in the studied area, this is unlikely for several reasons. Firstly, the majority of all oyster habitats have been privately owned for more than 300 years and oysters cannot be harvested without permission. Secondly, habitat destructive harvest methods such as dredging are prohibited, and harvest is only done manually by divers. Lastly, the overall harvest of the entire Swedish oyster population is roughly 0.25%, totaling ~10 tons (Swedish Agency for Marine & Water Management, 2018). Although, in individual local fishing areas, fishing could have an effect, this is unlikely to be reflected in samples and in the resulting models.

The observed increased importance of salinity and exposure for models of the distribution of dead shells is not fully matched by an alteration in partial dependence suggesting that increased importance is not entirely caused by changes in adaptation to different salinities or exposure levels. However, there are a tendency toward a shift toward slightly lower salinities and a decreased importance of medium exposed sites for the living assemblages. One potential explanation to this increased importance could be that the dissolution rates of *Ostrea edulis* shells are altered by salinity and exposure. Another explanation would be that shells of oysters growing in lower salinities are thinner due to higher cost of calcification (Malone & Dodd, 1967; Waldbusser, Voigt, et al., 2011) and thus dissolved faster upon death of the oyster. This increased cost is partly a result of lower salinity causing reduced Ca^2+^ concentration and lower levels of total inorganic carbon in the water (Hofmann et al., 2009; Mook & Koene, 1975). However, this is a less likely explanation, as this would have resulted in changed patterns in the partial dependence plots between the EOS and LO models, which is not observed (Figure 4). As the oyster shell is a dynamic resource subject to a number of processes causing degradation, it is most likely a combination of several processes explaining this observed difference and untangling these are beyond the scope of this study. One candidate process is increased water turbidity and local resuspension which are known to affect the growth of *O*. *edulis* with decreased growth, as the beneficial effects of resuspension (e.g., increased food supply) are inhibited by increased resuspension due to decreased ingestion and dilution of food with inorganics (Grant et al., 1990) and inhibiting recruitment (Moore, 1977).

The reason behind the observed pattern (Figure 6) of high frequency of dead assemblages in mainly two areas is currently unknown and needs further studies. Hypothesis about the causes includes loss of genetic variability inflicting weaker resilience toward infrequently occurring environmental events and diseases. Another would be local outbreaks of diseases or parasitic events affecting local populations. One interesting note is that the two areas with highest frequency of sites with dead assemblages are found at or close to the outer limits of the general occurrences of high‐density sites of the European flat oyster on the Swedish west coast, potentially favoring hypothesis on changing genetic resilience. It could also indicate that the spatial distribution of this fringe population of *O*. *edulis* is more affected than previously thought. This observed loss of high‐density habitats of *O*. *edulis* sites is in line with recent observations on decline and loss of another filter‐feeding bivalve (*Mytilus edulis*) areas along the Swedish coast (Baden et al., 2021). This conformity might indicate a more general change in the environmental conditions along the Swedish coast affecting, for example, the food supply for filter‐feeding bivalves with declining populations as a result. Maybe an unexpected negative result of a largely successful work on decreasing eutrophication in the area?

Species distribution models are an increasingly important tool in conservation and management (Hao et al., 2019). Using several models combined in an ensemble modeling approach may be a safer and more reliable method that can overcome uncertainty in model selection, thus further improving the reliability of the predicted spatial distribution.

Using species distribution models to further evaluate the spatial distribution of living and dead assemblages might be a possible way to estimate recent change in potential spatial distribution for larger areas. However, its interpretations require a clear distinction between potential and realized distributions (see Soberón, 2007). Using, as in this study, field data on living and dead shells will give models on the potential distribution (i.e., places where the species could live), while obtaining the actual distribution (realized distribution) requires a different full covering sampling approach which is most often not feasible for larger areas. Both potential and realized distribution refer to a specific period in time. While models of living oysters clearly represent the present distribution, the use of dead shells allows inferences about its past distribution. The exact age of this past distribution is, however, difficult to determine because of variability in the rate of shell degradation in different environments. The recent change in spatial distribution, mainly from a slight change in depth distribution, can have implications for restoration and for management, conservation, and sustainable use of oyster beds on the Swedish west coast, even though uncertainty caused by the potential transportation of dead shells from its origin needs to be included in such implications. However, this transport is not well known and thus difficult to account for. But, due to the rather rapidly decrease in energy with depth, it seems unlikely that wave action would cause strong influence in the distribution of dead shells due to movement of the relatively heavy oyster shells (~0.85 g cm^3^) in most of the investigated area. However, in cases of applying the demonstrated technique for species with lighter shells, shells more likely to be transported by wave energy, such considerations are increasingly important. Furthermore, the distinction between areas containing empty shells only and those with both living and dead help identify areas where there are no longer living oysters but where they previously thrived. However, modeled estimates of areal extents of dead and living oysters are very sensitive to cut‐off values (Table 3, Figure 5). Because the optimal cut‐off for living and dead oysters (based on a data‐driven approach using Youdens index) differs strongly (LO ≈ 0.1 and EOS ≈ 0.3), areas were potential not easily comparable between the two models. While it might be argued that the main concern in this context of this study is error due to false negative, we also used a more “decision‐analytic approach” (Steyerberg et al., 2011) where cut‐offs values were set equal for both models. This allowed estimation of areal extent of living and dead assemblages plus overlapping areas under conditions where the criteria for classification were equal. Therefore, due to the sensitivity of predicted areal extent to cut‐off values and the approaches used, caution is needed when evaluating and drawing conclusions from comparisons of models, and the conclusions might change depending on either data‐driven approach or more decision‐analytic approach is used.

Prediction based on models relies on full‐covering spatial data of all the predictors used, which are not always available. Naturally, several other environmental parameters such as pH, temperature, food availability, and gravel content in sediment (Bayne, 2017; Bergström et al., 2021; Cano et al., 1997; Davis & Calabrese, 1969; Laing & Spencer, 2006; Laing et al., 2005; Pardo et al., 2016) have been shown to potentially provide further knowledge about the causes of the species distribution. However, lacking full covering maps of these and many other potentially important environmental parameters limits their potential use in predictions of spatial distribution in the study area and many other areas. Developing maps or predictions of potentially important variables could in the future potentially provide important information for improved predictive species distribution models such as the one presented in this study.

This study of potential recent change in oyster areas along the Swedish west coast contributes to the objectives of the OSPAR Convention of Protection of the Seas (OSPAR, 2010), Habitat Directive (Directive 92/43/EG; Council of the European Union, 1992), and the Marine Strategy Framework Directive (MSFD; European Commission, 2008) by providing valuable information on potential recent changes in distribution of oyster reef areas on the Swedish west coast, thus providing valuable information for the successful preservation and restoration of these areas. Furthermore, it demonstrates a potential method to identify short‐term (decades) change in distribution range using dead assemblages in cases where reliable past distribution data are missing.

## AUTHOR CONTRIBUTIONS

**Per Bergström:** Conceptualization (lead); Formal analysis (lead); Methodology (lead); Writing – original draft (lead); Writing – review & editing (equal). **Linnea Thorngren:** Data curation (lead); Funding acquisition (supporting); Writing – original draft (supporting); Writing – review & editing (equal). **Mats Lindegarth:** Funding acquisition (lead); Methodology (supporting); Resources (lead); Writing – original draft (supporting); Writing – review & editing (equal).

## CONFLICT OF INTEREST

The authors declare that the research was conducted in the absence of any commercial or financial relationships that could be construed as a potential conflict of interest.

## Data Availability

All data from Thorngren et al. (2019) are available through the Open Science Framework https://osf.io/jgpxw/?view_only=d070b45802a4426da028efffde3d0f76, while additional data from Bergström et al. (2021) are available through the Open Science Framework https://osf.io/3agqp/?view_only=f2287b4c2cb041aeaca04cf66c560103.
